# Investigating SARS-CoV-2 breakthrough infections per variant and vaccine type

**DOI:** 10.3389/fmicb.2022.1027271

**Published:** 2022-11-24

**Authors:** Jozef Dingemans, Brian M. J. W. van der Veer, Koen M. F. Gorgels, Volker Hackert, Casper D. J. den Heijer, Christian J. P. A Hoebe, Paul H. M. Savelkoul, Lieke B. van Alphen

**Affiliations:** ^1^Department of Medical Microbiology, Infectious diseases and Infection prevention, Care and Public Health Research Institute (CAPHRI), Faculty of Health, Medicine and Life Sciences, Maastricht University Medical Center (MUMC+), Maastricht, Netherlands; ^2^Department of Sexual Health, Infectious Diseases and Environmental Health, South Limburg Public Health Service, Heerlen, Netherlands; ^3^Department of Social Medicine, Care and Public Health Research Institute (CAPHRI), Faculty of Health, Medicine and Life Sciences, Maastricht University, Maastricht, Netherlands

**Keywords:** SARS-CoV-2, breakthrough infections, whole-genome sequencing, variants, vaccine types

## Abstract

Breakthrough SARS-CoV-2 infections have been reported in fully vaccinated individuals, in spite of the high efficacy of the currently available vaccines, proven in trials and real-world studies. Several variants of concern (VOC) have been proffered to be associated with breakthrough infections following immunization. In this study, we investigated 378 breakthrough infections recorded between January and July 2021 and compared the distribution of SARS-CoV-2 genotypes identified in 225 fully vaccinated individuals to the frequency of circulating community lineages in the region of South Limburg (The Netherlands) in a week-by-week comparison. Although the proportion of breakthrough infections was relatively low and stable when the Alpha variant was predominant, the rapid emergence of the Delta variant lead to a strong increase in breakthrough infections, with a higher relative proportion of individuals vaccinated with Vaxzevria or Jcovden being infected compared to those immunized with mRNA-based vaccines. A significant difference in median age was observed when comparing fully vaccinated individuals with severe symptoms (83 years) to asymptomatic cases (46.5 years) or individuals with mild-to-moderate symptoms (42 years). There was no association between SARS-CoV-2 genotype or vaccine type and disease symptoms. Furthermore, the majority of adaptive mutations were concentrated in the N-terminal domain of the Spike protein, highlighting its role in immune evasion. Interestingly, symptomatic individuals harbored significantly higher SARS-CoV-2 loads than asymptomatic vaccinated individuals and breakthrough infections caused by the Delta variant were associated with increased viral loads compared to those caused by the Alpha variant. In addition, we investigated the role of the Omicron variant in causing breakthrough infections by analyzing 135 samples that were randomly selected for genomic surveillance during the transition period from Delta to Omicron. We found that the proportion of Omicron vs. Delta infections was significantly higher in individuals who received a booster vaccine compared to both unvaccinated and fully vaccinated individuals. Altogether, these results indicate that the emergence of the Delta variant and in particular Omicron has lowered the efficiency of particular vaccine types to prevent SARS-CoV-2 infections and that, although rare, the elderly are particularly at risk of becoming severely infected as the consequence of a breakthrough infection.

## Introduction

Since its discovery in December 2019 (Li et al., 2020), SARS-CoV-2 has infected more than 600 million people and caused about 6.5 million deaths as of October 11, 2022 (WHO, 2022). Although SARS-CoV-2 has a limited mutation rate of about two mutations per month (Duchene et al., 2020), which can be attributed to its 3′–5′ exonuclease proofreading activity (Minskaia et al., 2006), its rapid expansion across the globe has led to the emergence of numerous variants (Harvey et al., 2021; Peacock et al., 2021). Whereas the majority of variants initially harbored mutations outside the gene encoding the spike protein, a number of variants have emerged with mutations in the spike protein that affect infectivity or immune evasion. For example, the mutation leading to the D614G substitution in the spike protein emerged soon after SARS-CoV-2 spread to Europe at the beginning of the first wave (Korber et al., 2020). This mutation greatly affected the fitness of SARS-CoV-2 by stabilizing the open conformation of the spike protein, enhancing viral infectivity, and leading to dominance of this variant across the globe (Korber et al., 2020; Zhang et al., 2020; Plante et al., 2021). Further into the pandemic, a number of variants of concern (VOC) have emerged that harbor mutations in the receptor binding domain (RBD) of the spike protein, enhancing the affinity for the human angiotensin-converting enzyme 2 (ACE2) receptor (Ramanathan et al., 2021) or leading to evasion of the immune system (Hoffmann et al., 2021). In November 2020, the Alpha (B.1.1.7) variant was first reported as it quickly gained dominance in the UK, imposing a great burden on the healthcare system due to increased infectivity and slightly heightened mortality (Frampton et al., 2021; Davies et al., 2021a,b). Later, two other VOC (Beta and Gamma) were identified that exhibited significantly reduced neutralization by monoclonal antibodies or sera from recovered or vaccinated individuals (Weisblum et al., 2020; Chen et al., 2021; Geers et al., 2021; Harvey et al., 2021; Hoffmann et al., 2021; Planas et al., 2021; Wall et al., 2021; Wang Z. et al., 2021; Zhou et al., 2021). At the end of 2020, the Delta variant was identified in India, harboring the L452R and T478K mutations in RBD of the spike protein, showing moderately reduced neutralization and increased transmissibility compared to Alpha (Liu et al., 2021; Lopez Bernal et al., 2021; Planas et al., 2021; Wall et al., 2021). Finally, the Omicron (B.1.1.529) variant emerged in November 2021 and showed an unusually high number of mutations in the Spike protein that allowed this variant to efficiently escape from neutralizing antibodies in parallel with enhanced infectivity (Chen et al., 2022; Hu et al., 2022; Kurhade et al., 2022; Lyngse et al., 2022; Syed et al., 2022; Xia et al., 2022; Zou et al., 2022b).

Although many studies have assessed the potential of these variants to evade the immune system *in vitro*, only a limited number of studies have looked into the distribution of variants that can cause infections in fully vaccinated individuals. Monitoring these so-called post-vaccination “breakthrough variants” is imperative since they may signal widespread reduced vaccine efficacy early on. In this study, we have compared the occurrence of variants causing infections in 378 fully vaccinated individuals to the prevalence of variants that were identified by regional surveillance of SARS-CoV-2 in the South Limburg region of the Netherlands from January to July 2021. Furthermore, we investigated breakthrough infections during the transition period from the Delta to the Omicron era to investigate the role of the Omicron variant in causing breakthrough infections.

## Materials and methods

### Case definition

In this study, cases were defined as patients who were fully vaccinated against SARS-CoV-2 [≥14 days post-second dose of Comirnaty (mRNA-based vaccine; Pfizer-BioNTech), Spikevax (mRNA-based vaccine; Moderna), or Vaxzevria (Adenovirus vector-based vaccine; AstraZeneca) or ≥ 14 days post 1 dose of the Jcovden vaccine (Adenovirus vector-based vaccine; Janssen)] who had onset of COVID-19 related symptoms and subsequently tested positive by real-time PCR (RT-PCR) or antigen test for SARS-COV-2.

Cases were defined as symptomatic if they reported COVID-19 related symptoms, including common cold symptoms (nasal cold, runny nose, sneezing, or sore throat), cough, elevated temperature or fever (temperature > 38°C), loss of taste or smell, diarrhea, nausea, fatigue, headache, and generalized pain. Cases were defined as asymptomatic if they reported no COVID-19 related symptoms at the time of their positive test and developed no symptoms during the 7 day follow-up.

Symptomatic cases were further subdivided in mild–moderate and severe cases. All cases that were hospitalized or had a fatal outcome were classified as severe. All other symptomatic cases were classified as mild–moderate.

All post-vaccination breakthrough infections described were investigated by the Public Health Service South Limburg, the Netherlands. Cases or relevant staff members were contacted in order to gain missing data.

An overview of the proportion of individuals in South Limburg who received a booster vaccine and the type of the booster vaccine administered as of December 31, 2021 is shown in Supplementary Tables 1, 2, respectively. The great majority of individuals (95%) received the Comirnaty vaccine for their primary vaccination, while Spikevax, Vaxzevria, and the Jcovden vaccine were used to a much lesser extent at this stage. For booster vaccination, only the Spikevax (86%) and Comirnaty vaccine (14%) were administered. Thus, primary vaccine + booster vaccine combinations mainly consisted of either three doses of Comirnaty, three doses of Spikevax, two doses of Comirnaty + one dose of Spikevax, two doses of Spikevax + one dose of Comirnaty, two doses of Vaxzevria + one dose of Spikevax, two doses of Vaxzevria + one dose of Comirnaty, one dose of Jcovden + one dose of Spikevax, or one dose of Jcovden + one dose of Comirnaty.

### Sampling and RT-PCR assay

Trained personnel collected combined nasopharyngeal/oropharyngeal swabs in viral transport medium. Samples were sent either to the Medical Microbiological Laboratory of Maastricht University Medical Centre (MUMC+; workflow 1) or Synlab Belgium (workflow 2) for laboratory confirmation of SARS-CoV-2 *via* RT-PCR assay.

#### Workflow 1: RT-PCR analysis at the MUMC+

Samples were collected and transported in viral transport medium (Mediaproducts, The Netherlands). For RNA extraction, 900 μl of clinical sample was mixed with 900 μl of Chemagic Viral Lysis Buffer (Perkin-Elmer) and RNA was extracted from samples using the Chemagic Viral DNA/RNA 300 Kit H96 (Perkin-Elmer) on the Chemagic 360 system (Perkin-Elmer). A multiplex RT-PCR was performed using the N1-gene and E-gene as targets, including the immediate early gene of mouse cytomegalovirus as an internal control (Supplementary Table 3). cDNA synthesis and PCR amplification were combined using the TaqPath™ 1-Step RT-qPCR Master Mix, CG (Applied Biosystems, United States). Thermal cycling was performed using the Quantstudio 5 Real-Time PCR System (Applied Biosystems, United States). Oligonucleotides were synthesized and provided by Biolegio (Netherlands; Supplementary Table 3). Only the CT-value obtained for the N1 target was reported.

#### Workflow 2: RT-PCR analysis at Synlab Belgium

Alternatively, RT-PCR analysis was performed at Synlab Belgium using the ORF1ab, S-gene, and N-gene as targets. CT-values were reported for all three targets.

### Sequencing of SARS-CoV-2-positive samples

Sequencing was performed using the PCR tiling of SARS-CoV-2 virus with Native Barcoding Expansion 96 (EXP-NBD196) protocol (Version: PTCN_9103_v109_revH_13Jul2020) of Oxford Nanopore technologies, with minor modifications and using the primers previously published by Oude Munnink et al. (2020). Briefly, the only modifications were extending the barcode and adaptor ligation steps up to 60 min and loading 48 samples per flow cell. Bioinformatic analysis was performed using an in-house developed pipeline MACOVID^1^ that is based on Artic v1.1.3. Pangolin lineages were assigned using the Pangolin COVID-19 Lineage Assigner web application on https://pangolin.cog-uk.io/.

Consensus sequences with >3,000 Ns were considered as low quality and therefore excluded for further analyses. However, consensus sequences <10,000 Ns with a complete spike protein sequence were included.

### Mapping of non-lineage defining mutations on SARS-CoV-2 spike trimer model

The pdb file of the model 7D3F of the structure of the SARS-CoV-2 (Wuhan-Hu-1 strain) spike trimer in closed conformation as determined *via* cryo-electron microscopy by Xu et al. (2021) (doi: 10.2210/pdb7DF3/pdb) was visualized using the UCSF Chimera protein viewer (Pettersen et al., 2004). Non-lineage defining mutations were highlighted in red using the labeling tool in the Chimera protein viewer.

### Statistical analysis

All statistical analyses were performed using GraphPad Prism 9.0.0 software (GraphPad, La Jolla, CA, United States). A Mann–Whitney test was used to compare CT-values from breakthrough infections with the Alpha variant vs. infections caused by the Delta variant. A Kruskal-Wallis test was performed to compare median age, time of positive test post second dose, and CT value between groups (symptoms or vaccine types). Dunn’s multiple comparison test was performed *post hoc* in case of a significant difference to identify which groups significantly differed. Fisher’s exact test was performed to investigate the relationship between SARS-CoV-2 genotype and symptoms or vaccination status, while a Chi square test was performed to investigate the relationship between vaccine type and symptoms.

### Medical ethical approval

All data were retrieved from regular infectious disease control activities and were deidentified. The Medical Review Ethics Committee of the Maastricht UMC+ confirmed that the Medical Research Involving Human Subjects Act (WMO) does not apply to the above mentioned study and that an official approval of this study by the committee is not required (METC reference number 2021-2838).

## Results

### Description of the overall study population

In total, 378 breakthrough infections were recorded in the South Limburg region of the Netherlands until the end of week 28 in 2021. This study population had a median age of 45 years, consisted for 63% of women and most individuals (62%) received two doses of the Comirnaty vaccine (Table 1). The majority of cases were detected *via* community screening, while 8% of cases were related to care facilities (4% residents + 4% employees) and 14 cases (3.7%) were related to outbreaks (Table 1; Supplementary Table 4). The majority of people (75%) experienced mild to moderate symptoms and there were two fatalities among the seven severe cases (Table 1; Supplementary Table 4).

**Table 1:** Characteristics of the study population.

	Mean	Median	Range
Age (years)	46	45	18–96
	N	%	
Sex			
Female	239	63.2	
Male	137	36.2	
Unknown	2	0.5	
Vaccine			
Comirnaty	234	61.9	
Spikevax	9	2.4	
Vaxzevria	60	15.9	
Jcovden	72	19.0	
Other	1	0.3	
Unknown	2	0.5	
Setting			
Healthcare professionals	18	4.8	
Care Facility (employees)	15	4.0	
Care Facility (residents)	15	4.0	
Community	297	78.6	
Other	33	8.7	
Outbreak	14	3.7	
Symptoms			
Asymptomatic	82	21.7	
Mild–Moderate	285	75.4	
Severe	7	1.9	
Unknown	4	1.1	
Fatalities	2	0.5	

### Evolution of breakthrough cases in function of time and per vaccine type

**Figure 1:**
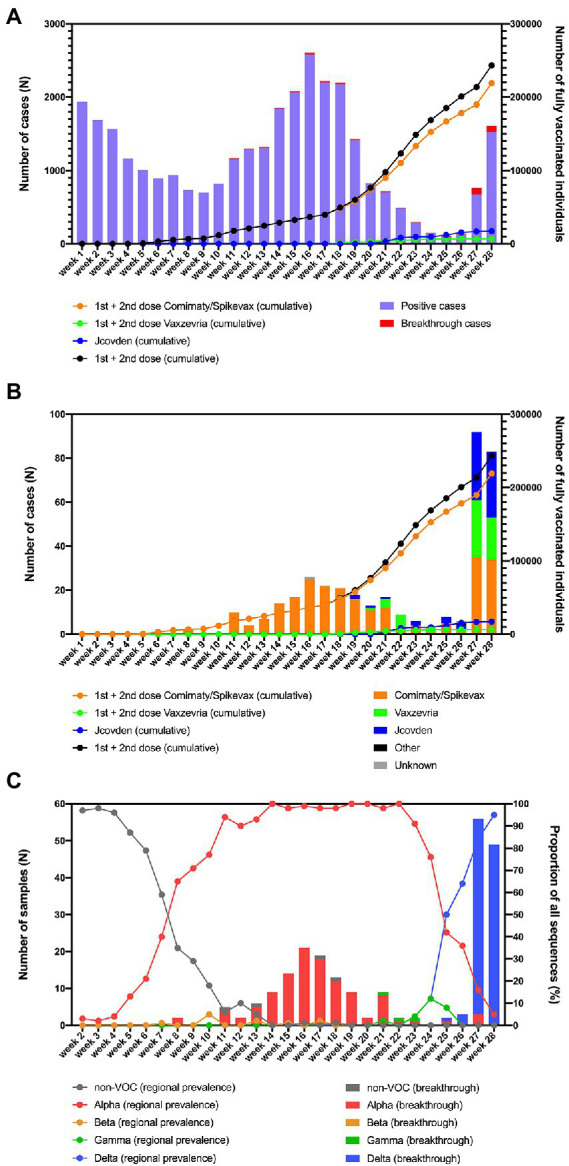
**(A)** Overview of the number of fully vaccinated individuals and breakthrough infections in function of time in the region of South Limburg. **(B)** Breakthrough infections per vaccine in function of time. **(C)** Evolution of the prevalence of Non-VOC and VOC lineages in the community (lines) as well as the distribution of variants causing breakthrough infections (bars) in function of time in the region of South Limburg. The numbers represented by bars are plotted on the left y-axis, while the numbers represented by dots/lines are plotted on the right *y*-axis.

The first individuals in the region of South Limburg received their second dose of the Comirnaty vaccine in week 4 and the number of people who were fully vaccinated steadily increased starting from week 6 (Figure 1A). The majority of people received an mRNA-based vaccine (mostly Comirnaty) and only starting from week 9, the first individuals were fully vaccinated with Vaxzevria, while the first fully vaccinated individuals who received the Jcovden vaccine started accumulating in week 21.

The first breakthrough infections were reported in week 8 and continually occurred starting from week 11, with a frequency ranging between 0.3 and 13.7% relative to the total number of infections (Figure 1A). The number of breakthrough cases steeply increased in week 27, after most COVID-19 measures were relaxed in the Netherlands and comprised disproportionally higher numbers of individuals who received Vaxzevria or Jcovden compared to the mRNA-based vaccines Comirnaty/Spikevax (Figure 1B). This was reflected by a considerably lower number of breakthrough cases relative to the number of administered vaccines for Comirnaty/Spikevax (0.14%) compared to Vaxzevria (0.89%) or Jcovden (0.46%; Table 2).

**Table 2:** Overview of breakthrough cases and administered vaccines per vaccine type.

	Comirnaty/Spikevax	Vaxzevria	Jcovden
*N* (breakthrough cases)	243	60	72
*N* (administered vaccines in week 26)	178,267	6,762	15,652
% breakthrough per vaccine^a^ [Table-fn tfn1]	0.14%	0.89%	0.46%

a The percentage of breakthrough cases per vaccine was calculated by dividing the total number of breakthrough cases recorded until the end of week 28 by the total number of administered vaccines in week 26, to ensure only vaccines received ≥14 days were included.

### The distribution of SARS-CoV-2 variants in fully vaccinated individuals generally coincides with their regional prevalence

From the 378 breakthrough cases that were identified from week 2 until week 28, 225 were successfully genotyped *via* whole-genome sequencing. The remaining samples had a Ct-value > 32 (*n* = 19), were unavailable for sequencing (*n* = 83), or did not comply with quality requirements of sequencing (*n* = 51).

At the same time, regional surveillance of SARS-CoV-2 variants was performed on a weekly base. The non-VOC genotypes, which mainly consisted of the B.1.177 and B.1.221 lineages that have been prominent in the region since the summer of 2020 (Hodcroft et al., 2021), were responsible for more than 50% of SARS-CoV2 infections up to week 7, after which the Alpha variant became dominant (Figure 1B). From this point, the frequency of Alpha infections rose quickly, constituting >90% of SARS-CoV-2 cases in week 11. In week 8, the first two breakthrough cases were identified, belonging to the Alpha variant (Figure 1C). Starting from week 11, the great majority of SARS-CoV-2 breakthrough infections were caused by the Alpha variant, with the exception of an occasional Gamma or a non-VOC infection (Figure 1C). The first breakthrough infection caused by the Delta variant was detected in week 25, when this variant was responsible for 50% of cases in the region and became dominant. As COVID-19 measures were relaxed in the Netherlands by the end of week 25, the number of breakthrough cases peaked during week 27 and 28, of which the great majority was caused by the Delta variant that was also dominant at that moment as determined *via* regional surveillance. The proportion of breakthrough infections caused by the Delta variant seems to be slightly higher than its share in genomic surveillance (100 vs. 64% in week 26, 95 vs. 84% in week 27, and 100 vs. 95% in week 28).

### Breakthrough cases infected with the Delta variant harbor a higher viral load compared to cases infected with the alpha variant

**Figure 2:**
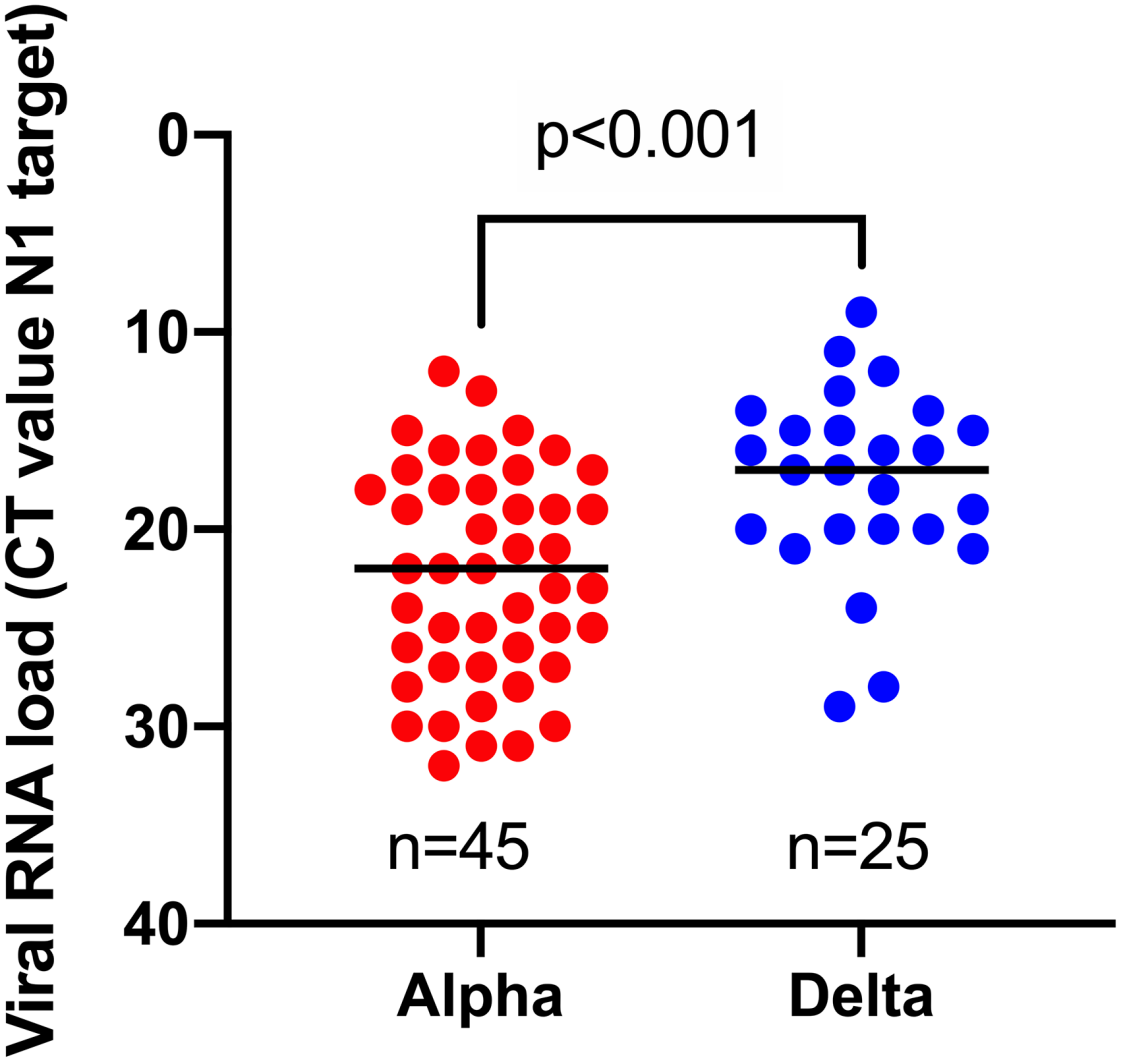
Comparison of viral RNA loads, obtained by the same workflow for samples harboring Alpha vs. Delta variant in breakthrough cases.

To investigate whether infections caused by the Delta variant are associated with increased viral loads compared to infections with the Alpha variant in fully vaccinated individuals, we compared the viral RNA load (based on the N1 target) as determined *via* a single workflow. A significantly higher viral RNA load (median CT-value = 17) was observed for breakthrough infections caused by the Delta variant vs. those caused by the Alpha variant (median CT-value = 22; Figure 2).

### Non-lineage defining mutations in the spike protein are mainly concentrated in the N-terminal domain among isolates causing breakthrough infections

**Figure 3:**
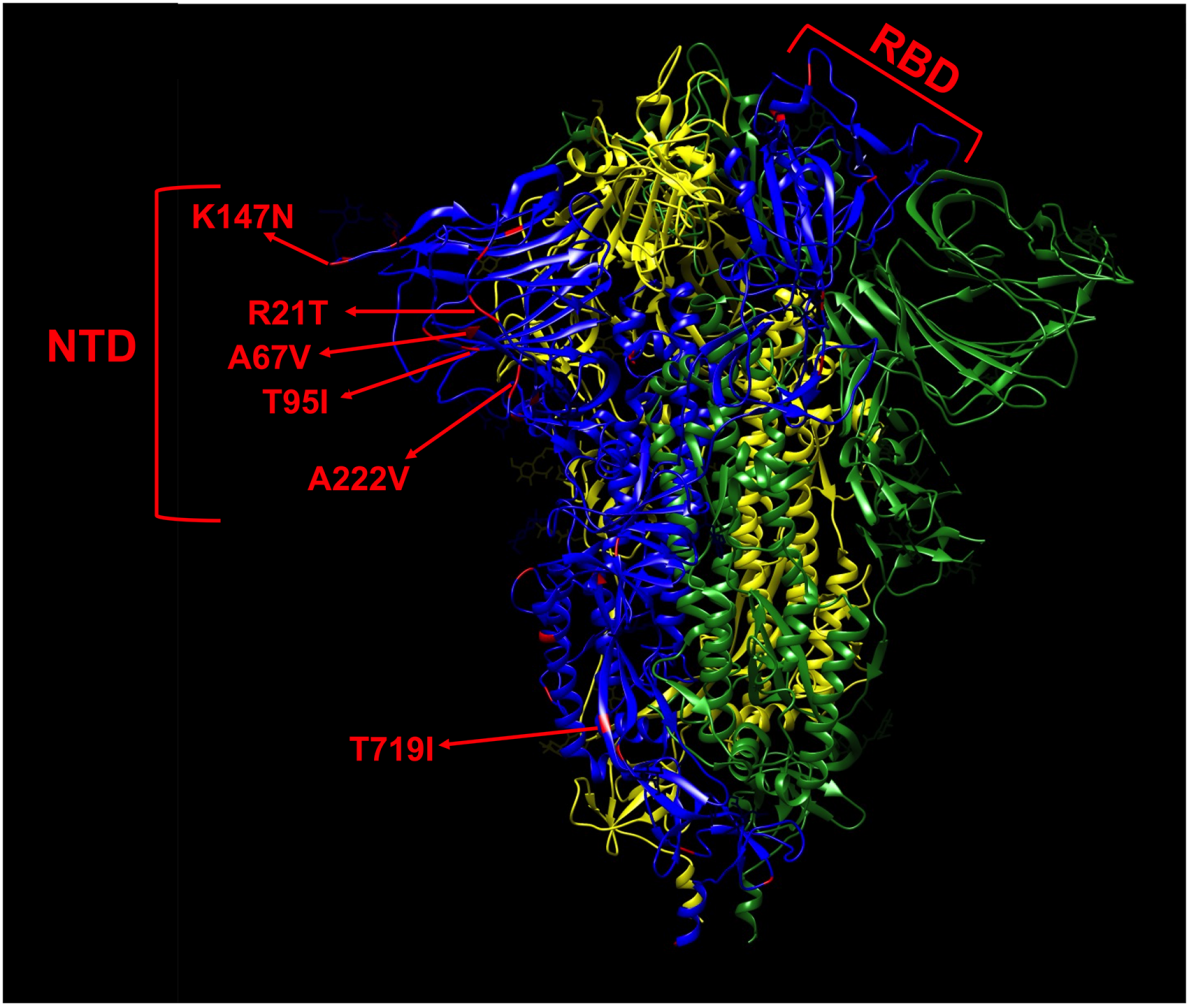
Mapping of non-lineage defining mutations (red) on one monomer (blue) of the spike protein. The most frequent mutations (>1%) are indicated by red arrows. The L5F, G1219V, I1232L, and L1265I mutations are not shown in this model due to their proximity to the termini as the model only comprises residues 14–1,147. The spike is shown in its closed state as a trimer of 3 monomers colored in blue, yellow, and green, respectively. NTD, N-terminal domain. RBD, receptor binding domain.

Of the 225 isolates that were sequenced, four were non-variants of concern (non-VOC), 112 Alpha variant, three Gamma, and 106 Delta variant. In total, 46 non-lineage defining mutations were identified in the spike protein of the 225 isolates that were sequenced (Table 2), of which nearly half (19/46) were located in the N-terminal domain, while only a small minority (5/46) was found in the receptor binding domain (RBD; Table 3; Figure 3), despite both domains being of comparable size. Nine of the 10 most frequent mutations (>1% frequency) occurred in the N-terminal domain (6/10) or in the C-terminal domain (3/10), and the two most observed mutations R21T (10.7%) and A222V (28.4%) were identified in at least two different variant backgrounds.

**Table 3:** Non-lineage-defining mutations in the spike protein found in this study.

Mutation	N^a^	Frequency (%)	*N* (%) in non-VOC	*N* (%) in Alpha	*N* (%) in Gamma	*N* (%) in Delta
L5F	8	3.6%	0 (0%)	8 (7%)	0 (0%)	0 (0%)
S12F	1	0.4%	0 (0%)	1 (1%)	0 (0%)	0 (0%)
L18F	1	0.4%	0 (0%)	1 (1%)	NA^b^	0 (0%)
R21T	24	10.7%	0 (0%)	1 (1%)	0 (0%)	23 (22%)
T22I	1	0.4%	1 (25%)	0 (0%)	0 (0%)	0 (0%)
T29I	2	0.9%	2 (50%)	0 (0%)	0 (0%)	0 (0%)
T29A	1	0.4%	0 (0%)	0 (0%)	0 (0%)	1 (1%)
A67V	7	3.1%	0 (0%)	0 (0%)	0 (0%)	7 (7%)
T95I	11	4.9%	0 (0%)	0 (0%)	0 (0%)	11 (10%)
K97Q	1	0.4%	0 (0%)	0 (0%)	0 (0%)	1 (1%)
K147N	3	1.3%	0 (0%)	3 (3%)	0 (0%)	0 (0%)
K150E	1	0.4%	0 (0%)	1 (1%)	0 (0%)	0 (0%)
M153I	1	0.4%	0 (0%)	1 (1%)	0 (0%)	0 (0%)
S221L	1	0.4%	0 (0%)	0 (0%)	0 (0%)	1 (1%)
A222V	64	28.4%	1 (25%)	0 (0%)	0 (0%)	63 (59%)
T250S	1	0.4%	0 (0%)	0 (0%)	0 (0%)	1 (1%)
T250I	1	0.4%	0 (0%)	0 (0%)	0 (0%)	1 (1%)
P251L	2	0.9%	0 (0%)	0 (0%)	0 (0%)	2 (2%)
A352S	1	0.4%	0 (0%)	1 (1%)	0 (0%)	0 (0%)
N439K	1	0.4%	1 (25%)	0 (0%)	0 (0%)	0 (0%)
K444N	1	0.4%	0 (0%)	1 (1%)	0 (0%)	0 (0%)
P479S	1	0.4%	0 (0%)	0 (0%)	0 (0%)	1 (1%)
A522S	1	0.4%	0 (0%)	0 (0%)	0 (0%)	1 (1%)
E583D	1	0.4%	0 (0%)	1 (1%)	0 (0%)	0 (0%)
S640F	1	0.4%	0 (0%)	1 (1%)	0 (0%)	0 (0%)
A653V	1	0.4%	0 (0%)	1 (1%)	0 (0%)	0 (0%)
A688V	1	0.4%	0 (0%)	1 (1%)	0 (0%)	0 (0%)
T719I	3	1.3%	0 (0%)	0 (0%)	0 (0%)	3 (3%)
K795R	1	0.4%	0 (0%)	1 (1%)	0 (0%)	0 (0%)
P812L	1	0.4%	0 (0%)	1 (1%)	0 (0%)	0 (0%)
D843N	1	0.4%	0 (0%)	1 (1%)	0 (0%)	0 (0%)
D936H	1	0.4%	0 (0%)	0 (0%)	0 (0%)	1 (1%)
V976F	1	0.4%	0 (0%)	0 (0%)	0 (0%)	1 (1%)
A1056V	1	0.4%	0 (0%)	1 (1%)	0 (0%)	0 (0%)
Q1071L	2	0.9%	2 (50%)	0 (0%)	0 (0%)	0 (0%)
G1085R	1	0.4%	0 (0%)	1 (1%)	0 (0%)	0 (0%)
I1115V	1	0.4%	0 (0%)	1 (1%)	0 (0%)	0 (0%)
S1147L	1	0.4%	0 (0%)	1 (1%)	0 (0%)	0 (0%)
T1160I	1	0.4%	0 (0%)	1 (1%)	0 (0%)	0 (0%)
G1219V	3	1.3%	0 (0%)	3 (3%)	0 (0%)	0 (0%)
V1228L	1	0.4%	0 (0%)	1 (1%)	0 (0%)	0 (0%)
I1232L	4	1.8%	0 (0%)	4 (4%)	0 (0%)	0 (0%)
M1237V	1	0.4%	0 (0%)	0 (0%)	0 (0%)	1 (1%)
V1264L	2	0.9%	0 (0%)	1 (1%)	0 (0%)	1 (1%)
L1265I	8	3.5%	0 (0%)	0 (0%)	0 (0%)	8 (8%)

a *N* = 225, consisting of 4 non-VOC, 112 Alpha, 3 Gamma, and 106 Delta isolates.

b Not applicable, this mutation is a defining mutation of the Gamma variant.

### People experiencing severe symptoms were significantly older than those who remained asymptomatic or experienced mild to moderate symptoms

**Figure 4:**
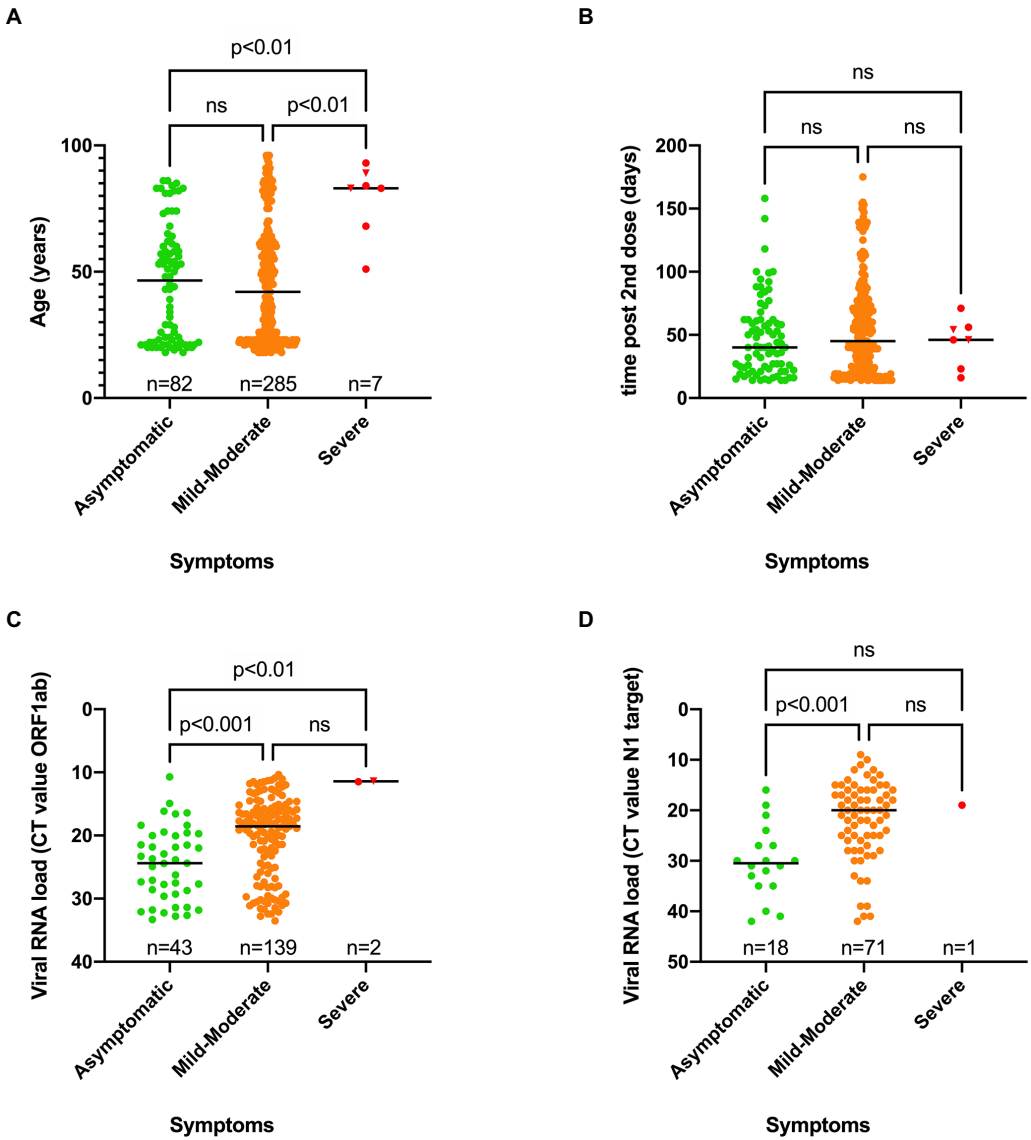
Scatter plots showing the distribution of age vs. symptoms **(A)**, time of positive RT-PCR test after receiving the second dose vs. symptoms **(B)**, Viral RNA load vs. symptoms for the ORF1ab target **(C)** or N1 target **(D)** as determined using two independent workflows. A Kruskal-Wallis test was performed to compare the median age **(A)**, median time of positive RT-PCR test after receiving the second dose **(B)** and the viral RNA load **(C,D)** between the three categories of symptoms. When a statistically significant result was obtained, Dunn’s multiple comparison test was performed *post hoc* to identify which groups significantly differed. ns, not significant. A fatal outcome was indicated by a reverse triangle symbol. The low number of severe cases might not provide enough statistical power to reveal statistically significant differences between this category and others.

A significant difference in median age was found between asymptomatic (46.5 years old) and severe symptomatic cases (83 years old) on one hand, and mild–moderate symptomatic (42 years old) and severe COVID-19 cases on the other hand (Figure 4A). Two out of seven severe SARS-CoV-2 infections had a fatal outcome. Nevertheless, the great majority of cases (75.4%) belonged to the mild–moderate category. There was no association between SARS-CoV-2 genotype and disease severity (Table 4).

**Table 4:** Contingency table of SARS-CoV-2 variant vs. symptoms.

*N* (Percentage of grand total)	Asymptomatic	Mild–Moderate	Value of p^*^
Alpha	22 (10.19%)	88 (40.74%)	0.28
Delta	15 (6.94%)	91 (42.13%)	

^*^Value of *p* was calculated using Fisher’s exact test.

When the time of the first positive test after receiving the second dose was plotted against disease severity for each case, no significant difference could be observed (Figure 4B).

However, a significant difference in viral RNA load was observed between the three different groups of symptoms, as higher viral RNA loads (reflected by a lower median CT value) were observed in the severe and mild–moderate categories compared to asymptomatic cases (Figures 4C,D).

### Relationship between vaccine type and viral load and time of positive test post vaccination and age

**Figure 5:**
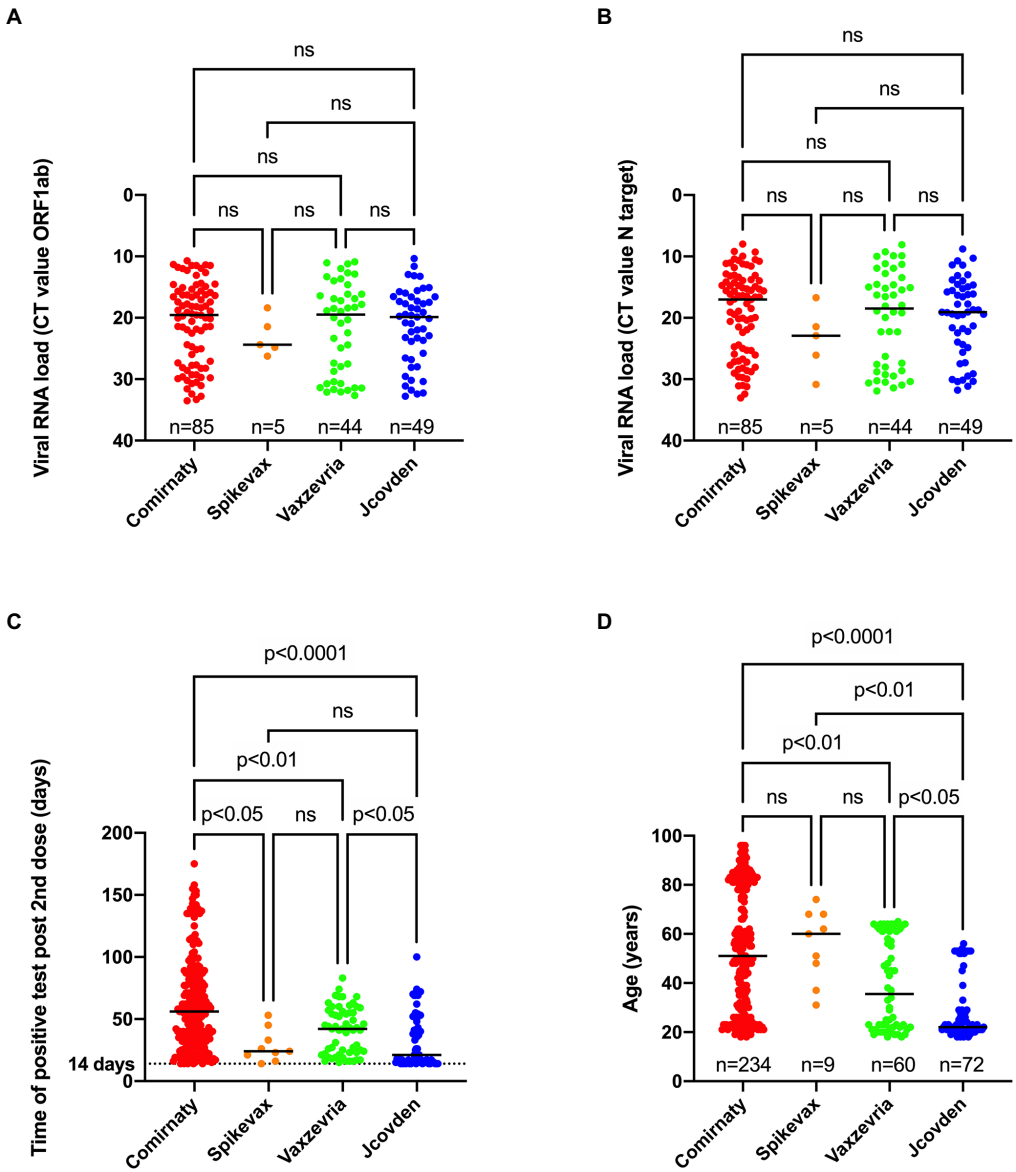
Relationship between type of vaccine used and viral RNA load obtained for ORF1ab **(A)**, N target **(B)**, time of positive SARS-CoV-2 test after receiving the second dose **(C)** or age **(D)**. A Kruskal-Wallis test was performed to compare median CT value for ORF1ab **(A)**, N target **(B)**, the median time of positive RT-PCR test after receiving the second dose **(C)** and the median age **(D)** between the four types of vaccines. When a statistically significant result was obtained, Dunn’s multiple comparison test was performed *post hoc* to identify which groups significantly differed. ns, not significant.

No significant difference in viral load could be observed when comparing the infections after Comirnaty, Spikevax, Vaxzevria, and Jcovden vaccinations (Figures 5A,B).

However, there was a significant difference in median time of the first positive test after receiving the second dose between the different vaccine types as people who received the Comirnaty vaccine tested positive later after being fully vaccinated compared to the other vaccine types (Figure 5C). In addition, people who received the Jcovden vaccine in our study received a positive result sooner after being fully vaccinated compared to people receiving the Vaxzevria vaccine.

Finally, individuals who received the Comirnaty vaccine were significantly older (median age 51 years old) than those who received the Vaxzevria (median age 35.5 years old) or the Jcovden vaccine (median age 22 years old; Figure 5D).

There was no association between vaccine type and disease severity (Table 5).

**Table 5:** Contingency table of vaccine type vs. symptoms.

*N* (Percentage of grand total)	Asymptomatic	Mild–Moderate	Value of p^*^
Comirnaty	53 (14.25%)	178 (47.85%)	0.96
Spikevax	2 (0.54%)	7 (1.88%)	
Vaxzevria	12 (3.23%)	48 (12.90%)	
Jcovden	15 (4.03%)	57 (15.32%)	

^*^Value of *p* was calculated using a Chi-square test.

### Proportion of infections caused by Omicron vs. Delta in unvaccinated, fully vaccinated, and boosted individuals

**Figure 6:**
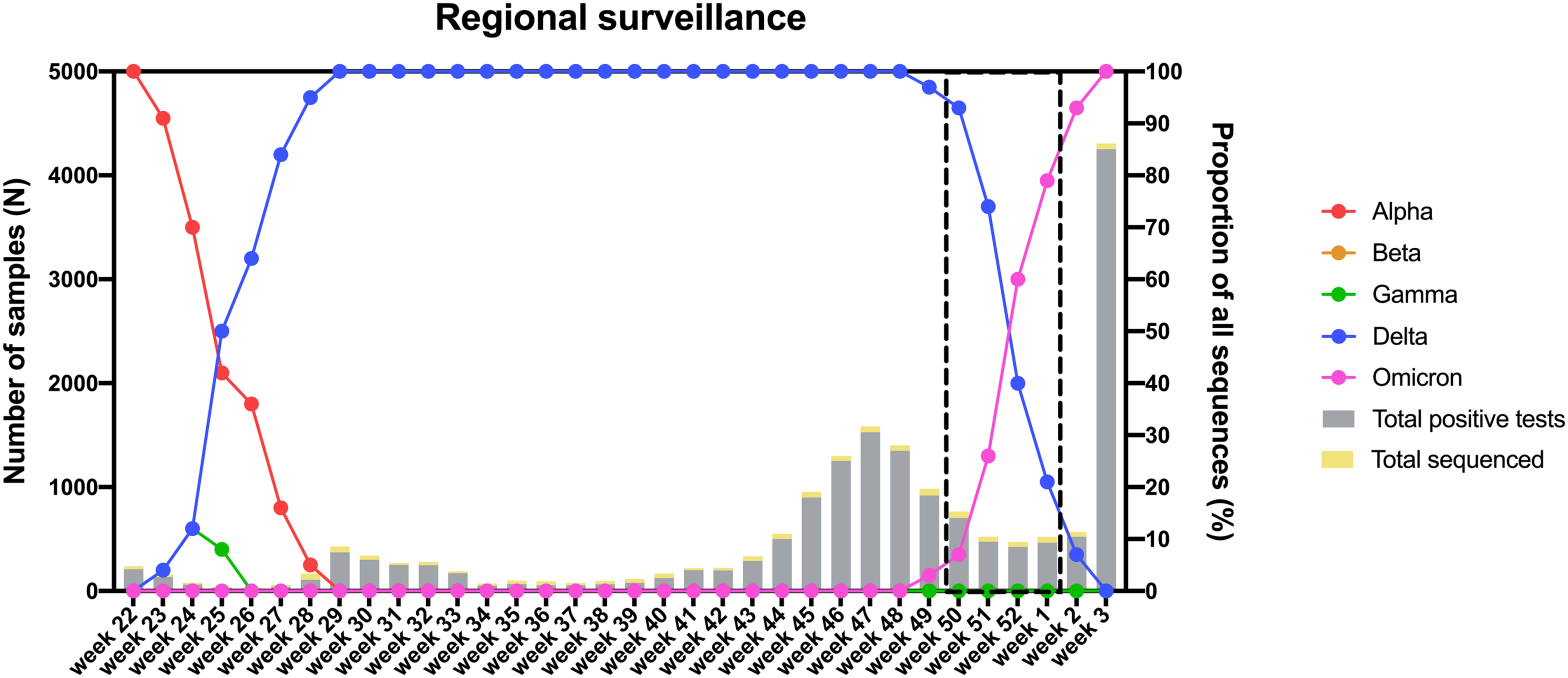
Distribution of circulating variants in the South Limburg region in time. Lines represent the relative proportion of each variant, while bars represent the total number of samples that were tested positive (gray) or were sequenced (yellow) in function of time. The transition period during which the Delta variant was replaced by Omicron is indicated by a dashed box.

As the Delta variant remained dominant until week 52 of 2021 (Figure 6) and numerous breakthrough infections were recorded in The Netherlands during the Delta era, we decided to focus on the transition period (week 50 of 2021 to week 1 of 2022) during which the Delta variant was replaced by Omicron to investigate the role of the latter variant in causing breakthrough infections. To study the proportion of breakthrough infections during this period, we stratified 135 samples that were randomly selected for genomic surveillance according to vaccination status. Of the 135 genotyped samples, 79 harbored Delta, while 56 harbored Omicron (Supplementary Table 5). Although there was no significant difference between the proportion of infections caused by Delta vs. Omicron when comparing unvaccinated to fully vaccinated individuals (Table 6), the proportion of Omicron vs. Delta infections was significantly higher in individuals who received a booster vaccine compared to both unvaccinated and fully vaccinated individuals (Tables 7, 8).

**Table 6:** Contingency table of SARS-CoV-2 variant vs. unvaccinated/fully vaccinated status.

*N* (Percentage of grand total)	Unvaccinated	Fully vaccinated	Value of p^*^
Delta	16 (13.56%)	60 (50.85%)	0.82
Omicron	10 (8.47%)	32 (27.12%)	

^*^Value of *p* was calculated using Fisher’s exact test.

**Table 7:** Contingency table of SARS-CoV-2 variant vs. unvaccinated/boosted status.

N (Percentage of grand total)	Unvaccinated	Boosted	Value of p^*^
Delta	16 (37.21%)	3 (6.98%)	0.0058
Omicron	10 (23.26%)	14 (32.56%)	

^*^Value of *p* was calculated using Fisher’s exact test.

**Table 8:** Contingency table of SARS-CoV-2 variant vs. fully vaccinated/boosted status.

N (Percentage of grand total)	Fully vaccinated	Boosted	Value of p^*^
Delta	60 (55.05%)	3 (2.75%)	0.0004
Omicron	32 (29.36%)	14 (12.84%)	

^*^Value of *p* was calculated using Fisher’s exact test.

## Discussion

In this study, we investigated breakthrough infections per variant and vaccine type in function of time in the South Limburg region of the Netherlands. We determined that prior to emergence of the Delta variant, breakthrough cases occurred at a continuously low frequency and the proportion of SARS-CoV-2 variants causing breakthrough infections corresponded well with the distribution of those variants in the region. However, both the number of positive cases and breakthrough infections steeply increased starting from week 27, most likely as a consequence of relaxation of COVID-19 measures and subsequent higher virus transmission rates in The Netherlands by the end of week 25. Furthermore, the proportion of breakthrough cases vs. total cases seemed increased. This rise started from week 25, when the Delta variant became dominant in the region. However, whether this increase in relative proportion of breakthrough infections is related to the rise of the Delta variant is difficult to determine since the number of fully vaccinated individuals also quickly accumulated during that period. Other studies have observed a similar impact of the Delta variant as for example in the Delaware valley, the Delta variant showed 3-fold enrichment in vaccine breakthrough cases compared to genomic surveillance data through summer 2021 (Marques et al., 2022). Furthermore, several studies have found that vaccine effectiveness for multiple vaccine types was significantly reduced against the Delta vs. Alpha variant (Cohn et al., 2021; Fowlkes et al., 2021; Lopez Bernal et al., 2021; Pouwels et al., 2021; Sheikh et al., 2021). For instance, a large study assessing breakthrough infections in frontline workers conducted by the CDC revealed that vaccine effectiveness dropped from 91% before predominance of the Delta variant to 66% when this variant became predominant (Fowlkes et al., 2021). A second study analyzing breakthrough infections in 780,225 US veterans found that vaccine effectiveness dropped from 87.9% in February when the Delta variant was absent to 48.1% in October when the Delta variant was predominant in the United States (Cohn et al., 2021).

In our study, we also noticed a disproportionate representation of administered vaccine types among breakthrough cases, as the mRNA-based vaccines (0.14%) led to fewer breakthrough infections, compared to the Jcovden (0.46%) or Vaxzevria vaccines (0.89%) relative to the number of administered vaccines. Since the number of fully vaccinated people with Vaxzevria and Jcovden only significantly started accumulating staring from week 21 and nearly half of the breakthrough cases were recorded between week 25 and week 28, this applies in particular to the Delta variant. A similar observation has been reported by another study in which it was shown that vaccine effectiveness for the Jcovden vaccine dropped from 86% in March to 13% in September, while the decline for the Spikevax (89% in March to 58% in September) and Comirnaty (87% in March to 43% in August) was much more modest (Cohn et al., 2021). Furthermore, the Washington DC Health department also found a higher proportion of breakthrough infections among people vaccinated with Jcovden (2.20%) as compared to Comirnaty (1.23%) or Spikevax (0.86%) relative to the number of complete vaccinations with the respective vaccine types between January and October (Washington DC Health Department, 2021). Finally, a third study showed that the Vaxzevria vaccine was 60% effective against infection with the Delta variant compared to 79% effectiveness for the Comirnaty vaccine, despite both vaccine types being 13% less effective compared to infection with the Alpha variant (Sheikh et al., 2021).

When comparing the distribution of SARS-CoV-2 variants retrieved from fully vaccinated individuals compared to the frequency of variants that were circulating in the entire population in the region of South Limburg, we found that breakthrough infections were caused by SARS-CoV-2 genotypes that were present in similar proportions during genomic regional surveillance. Before the Delta variant emerged, the great majority of breakthrough infections were caused by the Alpha variant with the exception of an occasional Gamma or a non-VOC infection. This is in line with data from the CDC in which 555 SARS-CoV-2 isolates from a total of 10,262 SARS-CoV-2 vaccine breakthrough infections were sequenced and the proportion of VOCs identified in breakthrough infections (64%) corresponded with their prevalence in the national genomic surveillance (70%) during the period between January 1 and April 30, 2021 (Fowlkes et al., 2021).

When the Delta variant emerged in the South Limburg region in week 23, the number of positive cases was low and this variant quickly became dominant in the following weeks when there was a surge in the number of positive cases, making it difficult to estimate what proportion of breakthrough infections are attributable to the Delta variant as compared to regional surveillance data. Nevertheless, the proportion of breakthrough infections caused by the Delta variant seems to be slightly higher than its share in genomic surveillance (100 vs. 64% in week 26, 95 vs. 84% in week 27, and 100 vs. 95% in week 28), although still being in line with our observations for the Alpha variant and other VOCs.

Marques and colleagues (Marques et al., 2022) found a much more pronounced enrichment of the Delta variant (3-fold) in SARS-CoV-2 breakthrough infections compared to its frequency in surveillance data in the Delaware valley. The fact that the time window from emergence to predominance of the Delta variant was wider (2 months vs. 1 month) in the Delaware study might explain this discrepancy.

When looking at the frequency of non-lineage defining mutations in the 225 isolates causing breakthrough infections in this study, it was found that the great majority of mutations were concentrated in the N-terminal and C-terminal domains of the spike protein. Among the three most frequent non-lineage defining mutations were the T95I (4.9%), R21T (10.4%), and A222V (28.4%) amino acid substitutions in the N-terminal domain, of which the latter two were detected in different variant backgrounds. It is known that mutations in the antigenic supersite of the N-terminal domain of the spike protein (residues 14–20, 140–158, and 245–264) can lead to reduced neutralization by a number of monoclonal antibodies (McCallum et al., 2021) For instance, mutation of residues 156–158 have been described in B.1.1.523 (van der Veer et al., 2021), being a variant under monitoring (VUM) and the Delta variant (Planas et al., 2021). Although the A222V and T95I mutations are in close proximity to the antigenic supersite residues, they have no effect on neutralization (McCallum et al., 2021). Nevertheless, the effect of these mutation in the Delta background on neutralization has not been determined yet and the T95I mutation is found in many VUM/VOI, including the B.1.1.318, Kappa, Iota, and Mu variants (Peacock et al., 2021; Uriu et al., 2021), a sublineage of the Delta variant (de Hoffer et al., 2022) as well as in the Omicron BA.1 and BA.3 sublineages (Zou et al., 2022a). Furthermore, the A222V mutation has emerged independently in the B.1.177 and two Delta sublineages, including the AY4.2 (“Delta Plus”) lineage that rapidly increased in frequency in the United Kingdom (de Hoffer et al., 2022). In addition, all A222V mutations in this study belonged to the AY.9 sublineage of the Delta variant that has also been associated with breakthrough infections in India (Gupta et al., 2021).

The finding that the majority of adaptive mutations are concentrated in the N-terminal domain corresponds well with recent data published by Zhang *et al*, showing that different variants tolerate multiple mutations causing structural rearrangements in their N-terminal domain that lead to immune evasion, while the overall structure of the RBD is strictly preserved, with mutations only being limited to a number of sites (Zhang et al., 2021).

Previously, we have shown that infections caused by the Delta variant are associated with higher viral loads compared to the Alpha variant in the total population (von Wintersdorff et al., 2022). Here, we found that this is also the case for a fully vaccinated population as a median difference of 5 CT-values was observed between infections caused by the Delta vs. Alpha variant. A similar difference in load between both variants was observed in the study of Blanquart et al., in which the CT values of 77 non-Delta (mostly Alpha) breakthrough infections were compared to 866 breakthrough cases infected with the Delta variant (Blanquart et al., 2021). A large study including 16,000 infected individuals during the Delta-dominant period in Israel found that fully vaccinated individuals harbored lower viral loads than unvaccinated individuals, but that the effect of vaccination on viral loads started declining after 2 months and completely vanished 6 months post vaccination (Levine-Tiefenbrun et al., 2021). However, the difference was restored after a booster dose of the Comirnaty vaccine. This indicates that viral loads found in vaccinated and unvaccinated individuals might be comparable in a relatively short amount of time post vaccination.

When looking at the distribution of disease symptoms in our study population, a significant difference in median age was observed between asymptomatic and severe (46.5 vs. 83 years) as well as mild–moderate and severe cases (42 vs. 83 years). Similar findings were reported in Belgium (Stouten et al., 2021) and the United States (Juthani et al., 2021), where the median age of hospitalized patients was 82 and 80.5 years, respectively. Only a low number of severe infections were observed in our study (seven cases; *ca.* 2%), although it has to be mentioned that our study only included the beginning of the Delta summer wave of 2021 in which the majority of infections were linked to the younger population. Nevertheless, the low percentage of severe cases in fully vaccinated individuals in this study corresponds well with national findings in The Netherlands, where only 5.7% of hospitalizations comprised fully vaccinated individuals and the vaccine efficacy against hospitalization was found to be very high against both the Alpha (94%) and Delta variant (95%; de Gier et al., 2021).

A number of hypotheses could explain the breakthrough infections causing mortality in the oldest age group. First of all, underlying conditions could have increased the vulnerability to SARS-CoV-2 infections in these individuals. Secondly, the humoral and cellular immune response triggered by the vaccines might not have been adequate to protect from symptomatic SARS-CoV-2 infection. Recently, it has been shown that there was a significant correlation between increased age and reduced antibody neutralization when sera from individuals vaccinated with Comirnaty were incubated with either wild-type, D614G, B.1.1.7, B1.351, or B.1.617.2 SARS-CoV-2 virus (Wall et al., 2021). Furthermore, a second study reported that a weaker T cell response was observed in older individuals following vaccination (Gallagher et al., 2021). It has been proposed that age-related thymic involution causes immunosenescence due to reduced T cell receptor (TCR) diversity as well as increased chronic inflammation in the elderly (Wang W. et al., 2021). Reduced TCR diversity could interfere with the ability to respond to novel antigens, while increased chronic inflammation could induce a cytokine storm (Wang W. et al., 2021).

No significant difference in time of positive test after the second dose could be observed between asymptomatic patients, patients with mild–moderate or severe symptoms in our study. However, it has been described that vaccine efficacy can drop over time as much as 22% for Comirnaty and 7% for Vaxzevria when comparing 14–90 days post receiving the second dose (Pouwels et al., 2021). The significant increase in age and time of the first positive test after receiving the second dose observed for the Comirnaty compared to the Vaxzevria and Jcovden vaccines can be explained by the fact that during the first 21 weeks mainly Comirnaty was used to inject mainly the elderly and healthcare workers, whereas the Jcovden vaccine was administered to young adults who received their second dose right before relaxation of COVID-19 measures when there was a spike in positive cases.

Interestingly, we found that symptomatic patients harbored significantly higher viral loads compared to asymptomatic people among breakthrough cases. A similar observation has been done by Shamier and colleagues in The Netherlands (Shamier et al., 2021) and by Blancqaert et al. in France (Blanquart et al., 2021). In agreement with the study of Shamier et al. (2021), no significant difference in viral load could be found when comparing the different types of vaccines that had been administered, despite the median CT-value for the few (*n* = 5) infections in Spikevax recipients being 5 units higher. A recent study has shown that higher neutralizing antibodies within a week before SARS-CoV-2 infection are associated with lower viral loads (Bergwerk et al., 2021). It would be interesting to see if the Spikevax vaccine is associated with lower viral loads compared to other vaccine types in a larger study of breakthrough infections, as higher antibody titers have been observed in people receiving two doses of Spikevax compared to two doses of Comirnaty (Steensels et al., 2021), most likely due to the >3-fold higher spike mRNA content. Most cases (75% based on the N1 target; 85% based on the ORF1ab target) in our study had a viral RNA load with a CT-value below 30, indicating that at the moment of sampling these individuals were likely to contain infectious viral particles. For example, Cuevas-Ferrando and colleagues (Cuevas-Ferrando et al., 2021) reported that nasopharyngeal samples below CT 30 harbored mostly intact viral particles. Another study found that the probability of viral recovery from samples between CT 27.5 and 30 was about 66% (Singanayagam et al., 2020). However, several studies (Shamier et al., 2021; Jung et al., 2022) have indicated that the probability of positive viral culture is lower in vaccinated individuals compared to unvaccinated individual at similar viral RNA loads, most probably due to the fact that a large fraction of viral particles are neutralized by antibodies in vaccinated subjects.

Finally, we also investigated the potential of the Omicron variant to cause breakthrough infections by looking at the 4-week time window when this VOC replaced Delta. We found that the Omicron variant was associated with a higher proportion of infections in boosted individuals compared to both unvaccinated and fully vaccinated individuals, however, we could not find a difference in the proportion of infections between Delta and Omicron when comparing individuals who completed their primary vaccination schedule to unvaccinated individuals. In contrast to this, other studies found that vaccine effectiveness was considerably lower for Omicron versus Delta when comparing both boosted and fully vaccinated individuals to unvaccinated subjects (Andeweg et al., 2022; Jalali et al., 2022; Lyngse et al., 2022; Tseng et al., 2022). This discrepancy could be explained by the effect of waning immunity since most individuals who completed their primary vaccination schedule during our study period received their second dose >6 months before the first positive PCR, a time after which vaccine protection against infection decreases significantly against both the Delta and Omicron variants (Gilboa et al., 2022; Goldberg et al., 2022). Because of this, any differences in the potential of Omicron vs. Delta in causing infections in fully vaccinated individuals might have been underestimated. In support of our findings, other studies found that the secondary attack rate in households infected with Omicron compared to Delta was significantly higher for boosted compared to fully vaccinated individuals (Jalali et al., 2022; Lyngse et al., 2022). Furthermore, one study found that the risk of transmission to other household members by primary cases infected with Omicron was more than 4-fold higher compared to Delta in boosted individuals vs. 1.44-fold in fully vaccinated individuals (Jalali et al., 2022). These findings, in combination with the observation that the viral load in fully vaccinated individuals was lower for Omicron compared to Delta (Puhach et al., 2022), indicate that other mechanisms such as immune evasiveness might explain the enhanced transmissibility of the Omicron variant.

Strengths of this study were the ability to monitor and compare breakthrough infections against the regional genomic surveillance over an extensive period, even before the first vaccines were administered as well as the regional follow-up of cases, allowing monitoring of the amount of breakthrough infections at a high resolution. A high proportion (60%) of all breakthrough cases were successfully sequenced further enhancing the resolution.

Limitations were the fact that the number of reported breakthrough infections are an underestimation of the effective number, due to asymptomatic or mild cases or because of severe cases in local hospitals that were not reported to the regional public health service. Furthermore, asymptomatic cases were only followed up for a limited amount of time, possibly leading to the underestimation of symptomatic cases. Thirdly, only samples from cases with sufficiently high viral loads could be sequenced (<CT 32), leading to a limited dataset. Additionally, not all samples were available so some bias could have been introduced if unavailable samples were different than available samples. Lastly, the lack of immunological data, which could help to understand how breakthrough infections occur and why certain individuals experience severe symptoms. Although the focus of our study was on vaccine breakthrough infections, sporadic cases with reinfections might have been missed. Also, people might have been unknowingly infected with SARS-CoV-2 during the first wave when there was a restrictive testing policy, which could not be shown due to the lack of serological data.

In summary, this study has investigated the distribution of breakthrough infections per vaccine type and variant over time in the South Limburg region of the Netherlands. It was found that breakthrough infections were more frequently observed in people who received Vaxzevria or Jcovden in comparison with recipients of the mRNA-based vaccines. Furthermore, the predominance of the Delta variant coincided with a rapid increase in breakthrough infections and severe cases were only observed in older individuals. Furthermore, we looked at breakthrough infections during the transition period from Delta to Omicron and found that the latter variant led to greater number of breakthrough infections in boosted individuals compared to Delta. Given that reduced vaccine effectiveness for both Delta and Omicron has been reported by this and other studies, highlights the need for adjusted vaccines that offer protection against the most recently circulating variants.

## Data availability statement

The datasets for this study can be found in the supplementary material of this article, while all SARS-CoV-2 sequences used in this study are publicly available on https://www.gisaid.org/. Documentation and source code are available from https://github.com/MUMC-MEDMIC/MACOVID under MIT license.

## Ethics statement

The studies involving human participants were reviewed and approved by Medical Review Ethics Committee of the Maastricht UMC+ (METC reference number 2021-2838). Written informed consent for participation was not required for this study in accordance with the national legislation and the institutional requirements.

## Author contributions

JD, BV, KG, VH, CHe, CHo, PS, and LA contributed to the conceptualization and the design of the study. JD drafted the manuscript, while BV, KG, and JD performed data analysis. KG performed investigation of breakthrough cases. BV and JD contributed to the visualization of the results. Interpretation and critical revision of the manuscript was done by all authors. All authors had access to the analyzed datasets, provided approval to submit the manuscript for publication, and also had access to verify the raw data. All authors contributed to the article and approved the submitted version.

## Conflict of interest

The authors declare that the research was conducted in the absence of any commercial or financial relationships that could be construed as a potential conflict of interest.

## Publisher’s note

All claims expressed in this article are solely those of the authors and do not necessarily represent those of their affiliated organizations, or those of the publisher, the editors and the reviewers. Any product that may be evaluated in this article, or claim that may be made by its manufacturer, is not guaranteed or endorsed by the publisher.

## Footnotes

^1^ https://github.com/MUMC-MEDMIC/MACOVID
