# Supplementary material for: Investigating SARS-CoV-2 breakthrough infections per variant and vaccine type
Source: Front Microbiol. 2022 Nov 24;13:1027271. doi: 10.3389/fmicb.2022.1027271 (PMC9729533; doi:10.3389/fmicb.2022.1027271)
Supplement: Supplementary file 1 [file Data_Sheet_1.PDF]

Supplementary Material

for

Investigating SARS-CoV-2 breakthrough infections per variant and vaccine type

Jozef Dingemans[1†]*, Brian M.J.W. van der Veer[1†]*, Koen M.F. Gorgels[2], Volker Hackert[2,3], Casper D.J. den Heijer[2,3], Christian J.P.A Hoebe[1,2,3], Paul H.M. Savelkoul[1], Lieke B. van Alphen[1].

Supplementary Table 1. Overview of the proportion of individuals in South-Limburg who completed their primary vaccination schedule and received a booster vaccine per age category as of 31 December 2021.

Supplementary Table 2. Overview of the proportion of each vaccine type used for primary and booster vaccination as of 31 December 2021.

Supplementary Table 3. Oligonucleotides used in this study.

Supplementary Table 4. Cohort metadata.

Supplementary Table 5. SARS-CoV-2 genomic surveillance samples obtained during the transition period from Delta to Omicron.
